# Effectiveness and Safety of Dabigatran Reversal with Idarucizumab in the Taiwanese Population: A Comparison Based on Eligibility for Inclusion in Clinical Trials

**DOI:** 10.3390/medicina59050881

**Published:** 2023-05-04

**Authors:** Jhih-Wei Dai, Chien-Ho Wang, Chan-Lin Chu, Shu-Chen Liao

**Affiliations:** 1 College of Medicine, Chang Gung University, Taoyuan City 333323, Taiwan; kent6101@gmail.com (J.-W.D.); m522@cgmh.org.tw (C.-H.W.);; 2 Department of Cardiology, Keelung Chang Gung Memorial Hospital, Keelung City 204201, Taiwan; 3 Department of Emergency Medicine, Keelung Chang Gung Memorial Hospital, Keelung City 204201, Taiwan; 4 Department of Neurology, Jen-Ai Hospital, Dali Branch, Taichung City 412224, Taiwan; 5 Master of Science Degree Program in Innovation for Smart Medicine, Chang Gung University, Taoyuan City 333323, Taiwan

**Keywords:** idarucizumab, dabigatran, reversal agents

## Abstract

*Background and Objectives:* The effectiveness and safety of idarucizumab for the reversal of the effects of dabigatran have been proven. However, there remains a paucity of literature comprehensively investigating outcomes in real-world patients. This is especially true when comparing patients who were eligible for inclusion in the RE-VERSE AD trial with patients who were ineligible. As the prescription of dabigatran has become increasingly popular, the generalizability of the results to real-world populations has come into question due to the broad variability of real-world patients receiving dabigatran. Our study aimed to identify all patients who were prescribed idarucizumab and examined how effectiveness and safety varied among those patients who were eligible and ineligible for the trial. *Materials and Methods*: This retrospective cohort study analyzed the largest medical database in Taiwan. We enrolled all patients who were prescribed and received idarucizumab from when it became available in Taiwan up until May 2021. A Total of 32 patients were included and analyzed, and they were further divided into subgroups based on their eligibility for inclusion in the RE-VERSE AD trial. Multiple outcomes were evaluated, including successful hemostasis rate, complete reversal efficacy of idarucizumab, 90-day thromboembolic events, intra-hospital mortality, and adverse event rate. *Results*: In our study, we found that 34.4% of real-world cases of idarucizumab use were ineligible for the RE-VERSE AD trials. The eligible group had higher successful hemostasis rates (95.2% vs. 80%) and anticoagulant effect reversal rates compared to the ineligible group (73.3% vs. 0%). The mortality rates were 9.5%, compared to 27.3% in the ineligible group. Few adverse effects (n = 3) and 90-day thromboembolic events (n = 1) were observed in either group. Among the ineligible cases, all acute ischemic stroke patients (n = 5) received definite, timely treatments without complications. *Conclusions:* Our study demonstrated the real-world effectiveness and safety of idarucizumab infusion for trial-eligible patients and all acute ischemic stroke patients. However, although it seems to be effective and safe, idarucizumab appears to be less effective in other trial-ineligible patients. Despite this result, our study provides further evidence for extending the applicability of idarucizumab in real-world scenarios. Our study suggests that idarucizumab can be a safe and effective option for reversing the anticoagulant effect of dabigatran, particularly for eligible patients.

## 1. Introduction

Dabigatran is widely used due to its clinical advantages over other anticoagulants, including its good tolerance, low potential for drug–drug interaction, predictable pharmacokinetics, and absence of need for frequent coagulation monitoring [1,2,3,4,5,6,7,8]. As the prescription of dabigatran has become gradually more popular, the reported annual major bleeding rate has varied from 2.71 to 3.36% [1]. To manage life-threatening situations associated with dabigatran, idarucizumab, a humanized dabigatran-specific monoclonal antibody with high affinity and specificity, was approved by the U.S. Food and Drug Administration and the European Medicines Agency in 2015 for patients suffering from uncontrolled bleeding or requiring emergency interventions. There are no contraindications for the administration of idarucizumab. [9,10]. The RE-VERSE AD study showed that idarucizumab is a rapid, safe, and lasting reversal agent in life-threatening scenarios, with an average hemostasis rate of 80.4% and 13.1% mortality, along with a 4.8% 30-day thromboembolic event rate [11]. The RE-VECTO study, a global surveillance program, further illustrated the global idarucizumab usage pattern in clinical practice and also demonstrated a low percentage of off-label use (<2%) [12].

After initial marketing, the effectiveness and safety of idarucizumab were evaluated through several studies, and these studies demonstrated a high rate of successful hemostasis with only a few thrombotic events or other serious adverse drug events (ADEs) [13,14,15,16,17,18]. The most commonly reported adverse reactions are headache and erythema [4,9]. However, since the guidance for the use of idarucizumab is based on indications from the preceding trials, the generalizability of idarucizumab has come into question due to the great variability of real-world patients receiving dabigatran [11,19]. Recently, a growing number of scenarios have seemed to benefit from idarucizumab use, such as ischemic stroke patients facing intravenous thrombolysis or intravenous tissue plasminogen activator treatment, which have been widely investigated [20,21,22,23,24,25,26]. Therefore, a better understanding of the breadth of applicability of idarucizumab is urgently needed to enhance the safety and well-being of dabigatran-treated patients.

There remains a paucity of comprehensive literature examining outcomes in real-world patients, especially in terms of comparing patients who would have been eligible for inclusion in the RE-VERSE AD trial with those who would have been ineligible. In our study, we aimed to examine all patients who were prescribed idarucizumab in one of the largest medical centers in Taiwan. Our goals were to determine how effectiveness and safety varied between patients who would have met the inclusion criteria of the RE-VERSE AD trial and those who would not have.

## 2. Methods

### 2.1. Study Design and Setting

We performed a retrospective and observational cohort study by analyzing the electronic medical records from the Chang Gung Research Database (CGRD). As one of the largest healthcare providers in Taiwan, Chang Gung Memorial Hospital annually handles an average of 8.6 million outpatient visits and around 370,000 admissions. The CGRD is the largest multi-institutional database in Taiwan, containing individual data from about 6% of the Taiwanese population [27,28]. This study was approved by the Chang Gung Medical Foundation Institutional Review Board, which waived the need for informed consent (IRB Number: 202101259B0).

### 2.2. Study Population, Eligibilities, and the Infusion Protocol for Idarucizumab

The inclusion criteria of our study were being more than 18 years old and receiving dabigatran. The only exclusion criterion was if patients did not actually receive the infusion following prescription. We enrolled all patients who were prescribed idarucizumab from when it became available in Taiwan, up until May 2021. Based on the inclusion criteria for the RE-VERSE AD trial, which can be retrieved from the study protocol of the trials, the potential trial eligibility of the individual patients who received idarucizumab was evaluated. This evaluation of eligibility was conducted by two independent reviewers (C.-H.W., J.-W.D.), whereby disagreements between the two reviewers were resolved in consultation with the senior author (S.-C.L.). We further divided all patients administered idarucizumab into three subgroups based on their eligibility category: Group A (uncontrollable or life-threatening bleeding, which is eligible for the RE-VERSE AD trials), Group B (emergent surgery or invasive procedures, which is eligible for the RE-VERSE AD trials), and Group C (ineligible for the RE-VERSE AD trials). As demonstrated in the RE-VERSE AD trial, adult patients who were receiving dabigatran and required surgery or an invasive procedure that could not be delayed for at least eight hours, or who were experiencing uncontrollable or life-threatening bleeding, were included. Patients who did not receive dabigatran, had minor bleeding, had elective surgery, or had a low risk of uncontrolled bleeding during the procedure were excluded. Following the inclusion and exclusion criteria of the RE-VERSE AD trial, we considered patients ineligible and classified them as Group C if they did not receive dabigatran or did not meet the specific or emergent conditions outlined in the trial. The definition of major and life-threatening bleeding was in accordance with the bleeding scale of the International Society of Thrombosis and Hemostasis (ISTH) [29]. Emergent surgery and invasive procedures were defined as interventions that could not be delayed by more than 8 h and situations where normal hemostasis was required [11].

The suitability of patients for idarucizumab infusion was evaluated by two physicians acting independently. Clinically, idarucizumab is administered intravenously as two consecutive infusions at 2.5 g/50 mL each, with at least a 10 min interval between each infusion.

### 2.3. Data Collection and Outcomes

We retrospectively collected the baseline characteristics for all patients prior to idarucizumab treatment. These parameters included age, sex, body weight, underlying comorbidity such as hypertension, diabetes mellitus, heart failure, previous ischemic stroke, acute coronary syndrome, and previous systemic embolism. We also recorded indications for dabigatran use, the daily dabigatran dosage, and laboratory data both before and after idarucizumab infusion, including creatinine clearance, activated partial thromboplastin time, international normalized ratio, hemoglobin level, and platelet count. Additionally, we calculated the CHA_2_DS_2_-VASc score, HAS-BLED score, and NIHSS score. To retrieve detailed information and the laboratory data of the selected patients, we retrieved electronic medical records from the Chang Gung Research Database (CGRD).

The primary safety outcome was defined as 90-day thromboembolic events, which comprised arterial (i.e., ischemic stroke, myocardial infarction, or peripheral vascular disease) or venous thromboembolism (i.e., deep vein thrombosis or pulmonary embolism). The secondary safety outcome was intra-hospital mortality, which was defined as death by any cause, documented in the medical record after idarucizumab infusion, during hospitalization. The third safety outcome was the rate of adverse events within 30 days. The adverse events included all adverse symptoms that were judged by the investigators to be related to idarucizumab.

The primary effectiveness outcome was the hemostasis rate, which was assessed and defined in accordance with the effectiveness of hemostasis, followed the International Society on Thrombosis and Hemostasis (ISTH) guidance, and varied depending on the situation [30]. The time of infusion of idarucizumab and the day of occurrence of thromboembolic events were both collected. The secondary effectiveness outcome was the reversal efficacy of idarucizumab. Complete reversal of anticoagulant effects was defined as the normalization of the activated partial-thromboplastin time (aPTT) after idarucizumab infusion. We chose aPTT over the diluted thrombin time (dTT) or the ecarin clotting time (ECT), as used in the RE-VERSE AD study, because the latter are not commonly used or available in the usual clinical settings in Taiwan [11]. Thus, we collected the blood sampling time and an aPTT value before and after infusion of idarucizumab. However, it is important to note that normal aPTT might not exclude on-therapy levels of dabigatran, and results should be interpreted cautiously [31].

### 2.4. Statistical Analysis

Descriptive statistics were used in this study. The categorical variables of the baseline demographic are presented in percentages (%) and the continuous variables are expressed as mean ± standard deviation (SD).

## 3. Results

### 3.1. Characteristics and Eligibility of Patients

We identified 47 patients from the Chang Gung Research Database (CGRD), all of whom were prescribed idarucizumab following its approval for use in Taiwan. However, 15 of these patients eventually did not receive idarucizumab. Among these 15 patients, 12 patients received catheter ablation for atrial fibrillation or left atrial appendage occlusion. Given the risk of bleeding during the procedure, which includes transseptal puncture and ablation for pulmonary vein isolation, operators usually prescribe preparations in case complications arise. One patient was misprescribed and did not receive an infusion, and one was using edoxaban and thus there was no administration of idarucizumab. The other patient was not infused with iarucizumab because no drug was available at that time. The study included the remaining 32 patients who received idarucizumab, with a mean age of 76.2 years and 46.9% being male. All patients received two consecutive infusions of 2.5 g/50 mL each (5 g of Idarucizumab). Of these 32 patients, 21 would have been eligible for the RE-VERSE AD trials. Their mean age was 79.6 years and 47.6% were male. More detailed baseline characteristics of the included patients are listed in Table 1.

**Figure 1:**
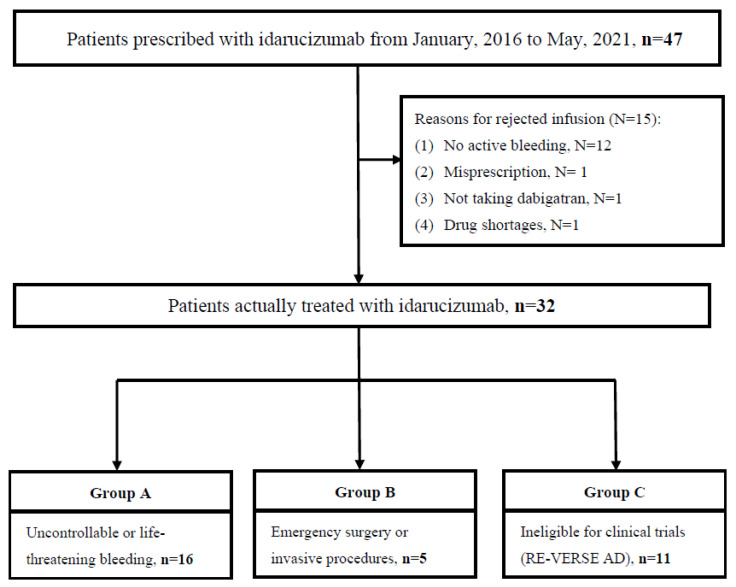
Study flowchart of the included patients.

We further divided the 32 patients into three subgroups. Group A contained 16 patients, including 8 with massive gastrointestinal bleedings, 4 with intracranial hemorrhages, 3 with symptomatic bleedings in a critical area, and 1 with massive hemoptysis. Group B comprised 5 patients, with 4 receiving surgeries due to intracranial hemorrhage and 1 undergoing emergent laparotomy. In Group C, patients were deemed ineligible for idarucizumab due to the reasons such as receiving intra-arterial thrombectomy (IA) or tissue plasminogen activator (TPA) infusion (45.5%, n = 5), not taking dabigatran (36.4%, n = 4), sepsis with disseminated intravascular coagulation but without active bleeding (9.1%, n = 1), and minor intramuscular bleeding (9.1%, n = 1). The study flowchart is presented in Figure 1.

### 3.2. The Safety and Effectiveness Outcomes of the Patients

Regarding the safety outcomes (Table 1 and Table 2), only one thromboembolic event with ischemic stroke was reported in the eligible group and none were reported among ineligible patients, with an overall thromboembolic event rate of 3.1% (1/32). The intra-hospital mortality rate was higher in the ineligible group (27.3%) compared to the eligible group (9.5%). Of all patients who received the infusion, only one patient (3.9%) with intracranial hemorrhage experienced rebleeding after receiving idarucizumab. The overall rate of adverse events was 9.4% (n = 3), with two patients in the eligible group experiencing delirium and aspiration pneumonia, and one patient in the ineligible group experiencing pneumonia.

In terms of the difference in effectiveness between eligible and ineligible patients (Table 1 and Table 2), the rate of successful hemostasis was higher in the eligible group (95.2% versus 80%). Furthermore, in the eligible group, 73.3% (11/15) of the patients achieved complete reversal of anticoagulant effects, with a median aPTT of 43.6 s prior to infusion of idarucizumab. However, in the ineligible group, none of the patients achieved complete reversal (0/10), with a median aPTT of 35.7 s before infusion. The detailed information is summarized in Table 3.

## 4. Discussion

Our study revealed that 34.4% of the real-world cases of idarucizumab use would have been ineligible for inclusion in the RE-VERSE AD trials. Our findings confirmed that idarucizumab is safe and effective for Taiwanese patients who meet the eligibility criteria of the RE-VERSE AD trials, as well as for acute ischemic stroke patients facing emergent interventions who would have been ineligible. On the other hand, our results also suggested that the safety profile of idarucizumab is comparable, but its effectiveness may be limited, in other patient groups who would have been ineligible for the trials.

In this retrospective study, we aimed to evaluate the effectiveness and safety outcomes of idarucizumab in an Asian population, stratified by eligibility for participation in the RE-VERSE AD trial. The eligible and ineligible groups had similar sex distribution, body weight, and daily dose of dabigatran, but the eligible group was older (79.6 vs. 69.5 years old) and had a higher previous systemic embolism rate (23.8% vs. 9.1%) and a higher baseline median aPTT value (43.2 vs. 35.7 s). The overall successful hemostasis rate among the potentially eligible patients was high (95.2%), with a considerable rate of complete reversal of anticoagulant effects (73.3%) and excellent safety outcomes, including a low thromboembolic event rate (4.8%), rebleeding rate (4.8%), intra-hospital mortality rate (9.5%) and adverse event rate (9.5%), compared to the original RE-VERSE AD trials. Thus, consistent with previous studies, idarucizumab generally provided instant and effective reversal of anticoagulant effects with few post-infusion side effects, regardless of race [11,13,15,16,17,32,33]. By contrast, among ineligible patients, the successful hemostasis rate (80%) and reversal of anticoagulant effects (0%) were less prominent, despite a low adverse event rate (9.1%), rebleeding rate (0%), and thromboembolic event rate (0%). Nonetheless, among these ineligible patients, dabigatran-treated acute ischemic stroke patients were able to safely receive intravenous thrombolysis or intravenous tissue plasminogen activator treatment after the infusion with idarucizumab.

For dabigatran-treated acute ischemic stroke patients, hemorrhagic transformation was the major concern before definite intervention such as intravenous thrombolysis or intravenous tissue plasminogen activator treatment. In our study, around half of the trial-ineligible patients were acute ischemic stroke patients who qualified for intravenous thrombolysis (n = 1, 20%) or intravenous tissue plasminogen activator treatment (n = 5, 100%). The median initial NIHSS score was 13.2, and all had uneventful courses except for one patient who developed subsequent aspiration pneumonia. The average time from the infusion to the definite intervention was 29 min. A recent systematic review of dabigatran-treated patients infused with idarucizumab before intravenous thrombolysis or intravenous tissue plasminogen activator demonstrated the effectiveness and safety of this therapeutic strategy. The review found favorable outcomes regarding the rate of hemorrhagic transformation and mortality compared to non-anticoagulated patients [21,23,26,34]. Our findings are comparable to the results of previous studies in which idarucizumab was not only a feasible therapeutic strategy but also saved valuable time for the subsequent definite treatments.

Our study also included other trial-ineligible patients from previous trials. A total of 11 patients were found to be ineligible for the study. Among these 11, 4 patients were not eligible due to not taking dabigatran (three were taking rivaroxaban, and one was taking apixaban), whereas 2 patients were not experiencing life-threating bleeding. In addition, 5 patients taking dabigatran received tissue plasminogen activator (TPA) for acute ischemic stroke. Despite the low case numbers, the findings in these patients are consistent with the fact that idarucizumab is the specific reversal agent for dabigatran—higher mortality rate (60%), lower successful hemostasis rate (80%), and low complete anticoagulant reversal rate (0%). Therefore, the assumption that this therapeutic strategy may have limited value in this non-qualified population is reasonable [14]. Further research is required to determine the efficacy of idarucizumab in this subset of patients, given the small sample size of our study.

Among the 47 patients who were prescribed idarucizumab, 12 (25.5%) were atrial fibrillation patients who underwent radiofrequency catheter ablation. The infusion of idarucizumab was not performed because there was no uncontrolled major bleeding during the intervention. Given the high risk of bleeding during the procedure, which involves transseptal puncture and ablation of the left atrium for pulmonary vein isolation, the anticoagulant strategy for patients with atrial fibrillation who undergo catheter ablation merits attention. Uninterrupted dabigatran is one of the preferred anticoagulant strategies, not only because it has fewer bleeding complications, but also because specific reversal agents are available [35,36,37,38,39]. The hemostasis rate of idarucizumab in an uncontrolled bleeding situation during catheter ablation is around 80%, with few adverse effects reported [38,40]. Thus, despite none of the patients who underwent radiofrequency catheter ablation actually being administered idarucizumab, uninterrupted dabigatran, with prepared idarucizumab on standby for emergency situations appears feasible in clinical settings which were not studied in the RE-VERSE AD trials.

In terms of adverse effects in real-world settings, the most common relevant side effects include delirium (7%), constipation (7%), pyrexia (6%) and pneumonia (6%) [14]. In comparison to the RE-VERSE AD trials where 23.3% (117/503) of the enrolled patients reported side effects, our study had a lower incidence, at 9.4% (3/32), with only one patient experiencing delirium and two patients developing aspiration pneumonia. Thromboembolic events were also a major concern due to the rebound effect. In the RE-VERSE AD trials, the thromboembolic event rate was 4.8% (24/503), whereas in our study, only a single event was found. This patient had atrial fibrillation and left middle cerebral artery infarction with hemorrhage transformation, in 2017, while taking dabigatran 150 mg twice daily to prevent ischemic stroke. In May 2021, the patient was prescribed idarucizumab due to left chronic subdural hemorrhage with midline shift (3.5 mm). Following treatment, the patient was discharged and did not use dabigatran or other anticoagulants after discharge. However, the patient experienced left-sided weakness and a recurrent ischemic stroke in the right temporo-occipital area and right subacute SDH with midline shift (10.2 mm), as revealed by a magnetic resonance imaging conducted on 23 June 2021. The patient received subdural drainage and recovered well without focal neurological signs. Eventually, the patient received dabigatran 110 mg twice daily again and refused left atrial appendage occlusion after discussion with a cardiovascular doctor. The time interval between the infusion of idarucizumab and the onset of right temporo-occipital ischemic stroke was 35 days. In our study, we found a lower rate of 90-day thromboembolic events, 3.1%. Additionally, a recent meta-analysis reported a pooled thromboembolic event rate of around 5.5% over 90 days in patients treated with a specific antidote [15,33]. Therefore, despite the proven safety record of idarucizumab, close monitoring for possible adverse effects is necessary in real-world settings.

Patients with atrial fibrillation commonly have coronary artery disease, and treating them with anticoagulants combined with antiplatelet therapy can be complex and challenging. Dual antiplatelet therapy (DAPT) is necessary for acute coronary syndrome, or stenting for coronary artery disease, and oral anticoagulants for stroke prevention are indicated in these patients due to a CHA_2_DS_2_-VASc score of at least 1, as well as the simultaneous presence of other cardiovascular risk factors. The 2021 European Heart Rhythm Association Practical Guide suggests that the use of direct oral anticoagulant (DOAC) combined with DAPT for up to 30 days may be advisable in patients with a high atherothrombotic risk, followed by a shift to therapy with DOAC combined with P2Y12 inhibitor for six months to one year [39]. However, the guideline also emphasizes the need to individualize the duration of combined therapy of DAPT and DOAC based on atherothrombotic and bleeding risk, as the risk of bleeding is expectedly elevated when anticoagulants are combined with antiplatelet therapy, creating a dilemma in clinical practice when the patient experiences life-threatening bleeding or requires emergency surgery. Additionally, the specific combination of drugs used in triple therapy may have an impact on the risk of bleeding complications [41]. To our knowledge, only two case reports have discussed the use of idarucizumab in this condition, and there are no trials addressing this issue [42,43]. Despite few case reports discussing the use of idarucizumab in patients with both atrial fibrillation and coronary artery disease, it may be a useful option in these patients who face life-threatening bleeding or require emergency surgery. Further research is needed to better understand how the composition of triple therapy affects the incidence and severity of bleeding complications and to evaluate the efficacy and safety of idarucizumab in this specific population.

The major strength of our study is that it provides comprehensive results for an idarucizumab-treated population and compares the effectiveness and safety outcomes with respect to patients’ trial eligibility, which has seldom been reported before. Relatively few studies have investigated the effectiveness and safety of idarucizumab in an Asian population. However, some limitations remain to our study. First, our study is retrospective, which means it could be subject to selection bias and confounding factors. Second, due to the relative infrequency of idarucizumab infusion, and despite utilizing the largest multi-institutional database in Taiwan, we were only able to enroll a smaller number of patients compared to previous trials. This limitation may restrict the generalizability of our findings and there may be differences in patient characteristics or treatment practices across different institutions. Third, the unknown duration between the last administration of dabigatran and the infusion of idarucizumab may have influenced our assessment of the effectiveness of idarucizumab. However, this aspect brings our study closer to real-world clinical conditions. Fourth, since our study lacked a control arm, it is not possible to make a direct comparison. As a result, the findings should be interpreted with caution. Fifth, in our study, the infusion rate of idarucizumab was not recorded in our medical records. Consequently, we were unable to gather additional information regarding the infusion rate and make comparisons to previous studies regarding safety and efficacy outcomes. Sixth, the timing of aPTT measurement varied in our study, with most of the data collected covering hours to days, or some data not being collected at all. In addition, some exact times of idarucizumab infusion were not available. The variability in the timing of aPTT measurements and the lack of exact timings of idarucizumab infusion made it difficult for us to assess the onset of the drug’s reversal effect, resulting in some inaccuracy in the determination of successful hemostasis rates. Finally, due to the nature of the study, several important issues were not addressed, such as choice of anticoagulants for resumption after the infusion.

## 5. Conclusions

In Taiwan, 34.4% of real-world cases of idarucizumab use would have been ineligible for participation in the initial safety and efficacy trials. However, our study has demonstrated the real-world effectiveness and safety of administration of idarucizumab among those who would have been eligible for the trials, as well as among acute ischemic stroke patients, regardless of their eligibility for the trials. In contrast, for trial-ineligible patients, although idarucizumab administration seems to be safe, it appears to be less effective. Our study provided further evidence for extending the applicability of idarucizumab in real-world scenarios.

## Figures and Tables

**Table 1:** The demographics and clinical data of patients who received idarucizumab.

Variable	All n = 32	Eligible for Trials n = 21	Group A n = 16	Group B n = 5	Group C n = 11	RE-VERSE AD, n = 503
Male, n (%)	15 (46.9%)	10 (47.6%)	8 (50%)	2 (40%)	5 (45.5%)	274 (54.5%)
Age, mean (SD, years old)	76.2 (11.5)	79.6 (9.6)	81.2 (9.3)	74.6 (9.8)	69.5 (12.5)	78
Body weight, median (SD, kg)	63.6 (14.2)	60.2 (11.9)	60.6 (11.8)	58.9 (13.8)	70.3 (16.6)	75
Comorbidity, n (%)						
Hypertension	19 (59.3%)	11 (52.4%)	10 (62.5%)	1 (20%)	8 (72.7%)	394 (78.3%)
Diabetes mellitus	10 (31.3%)	8 (38.1%)	5 (31.3%)	3 (60%)	2 (18.2%)	152 (30.2%)
Heart failure	11 (34.4%)	8 (38.1%)	6 (37.5%)	2 (40%)	3 (27.3%)	182 (36.2%)
Previous ischemic stroke	14 (43.8%)	8 (38.1%)	5 (31.3%)	3 (60%)	6 (54.6%)	47 (9.3%)
Acute coronary syndrome	3 (9.4%)	3 (14.3%)	2 (12.5%)	1 (20%)	0 (0%)	178 (35.4%)
Previous systemic embolism	6 (18.8%)	5 (23.8%)	5 (31.3%)	0 (0%)	1 (9.1%)	36 (7.2%)
Creatinine clearance, mL/min (%)						
≥80	9 (28.1%)	4 (19.0%)	3 (18.8%)	1 (20%)	5 (45.5%)	108 (21.5%)
30–80	20 (62.5%)	14 (66.7%)	10 (62.5%)	4 (80%)	6 (54.6%)	290 (57.6%)
<30	3 (9.4%)	3 (14.3%)	3 (18.8%)	0 (0%)	0 (0%)	91 (18.1%)
Dabigatran indications						
Atrial fibrillation	27 (84.4%)	19 (90.5%)	14 (87.5%)	5 (100%)	8 (72.7%)	478 (95%)
Systemic embolism	5 (15.6%)	4 (19.0%)	4 (25%)	0 (0%)	1 (9.1%)	9 (1.8)
CHA_2_DS_2_-VASc score, median	4.9	5.2	5.3	5.0	4.2	N/A
HAS-BLED score, median	2.7	2.9	3.2	1.8	2.5	N/A
Initial NIHSS score, median	13.5	15	N/A	15	13.2	N/A
Daily dose of dabigatran, n (%)						
150 mg twice daily	6 (18.8%)	5 (23.8%)	4 (25%)	1 (20%)	1/7 (14.3%)	151 (30%)
110 mg twice daily	22 (68.8%)	16 (76.2%)	12 (75%)	4 (80%)	6/7 (85.7%)	311 (61.8%)
Successful hemostasis, n (%)	24/26 (92.3%)	20/21 (95.2%)	15 (93.8%)	5 (100%)	4/5 (80%)	80.4%
Complete reversal of anticoagulant effects, n (%) ^a^	11/25 (44.0%)	11/15 (73.3%)	9/12 (75%)	2/3 (66.7%)	0/10 (0%)	N/A
Mortality, n (%) ^b^	5 (15.6%)	2 (9.5%)	2 (12.5%)	0 (0%)	3 (27.3%)	13.1%
Thromboembolic events, n (%) ^c^	1 (3.1%)	1 (4.8%)	0 (0%)	1 (20%)	0 (0%)	24 (4.8%)
Rebleeding rate, n (%)	1/26 (3.9%)	1/21 (4.8%)	1 (6.3%)	0 (0%)	0/5 (0%)	10 (2.0%)
Resumption of DOAC, n (%)	20 (62.5%)	14 (66.7%)	10 (62.5%)	4 (80%)	6 (54.5%)	N/A
Choice of DOAC after resumption, n (%)						N/A
Dabigatran	9 (28.1%)	5 (23.8%)	3 (18.8%)	2 (40%)	4 (36.4%)	N/A
Apixaban	4 (12.5%)	3 (14.3%)	2 (12.5%)	1 (20%)	1 (9.1%)	N/A
Edoxaban	2 (6.3%)	2 (9.5%)	2 (12.5%)	0 (0%)	0 (0%)	N/A
Rivaroxaban	2 (6.3%)	1 (4.8%)	1 (6.3%)	0 (0%)	1 (9.1%)	N/A
Other anticoagulants/antiplatelets	3 (9.4%)	3 (14.3%)	2 (12.5%)	1 (20%)	0 (0%)	N/A
Adverse side effects ^d^, n (%)	3 (9.4%)	2 (9.5%)	0 (0%)	2 (40%)	1 (9.1%)	117 (23.3%)
Laboratory data before idarucizumab						
aPTT, median (s)	43.6	42.3	52.9	33.7	35.7	N/A
Prolonged aPTT, n (%)	17 (53.1%)	16 (76.2%)	13 (81.3%)	3 (60%)	1 (9.1%)	372 (74.2%)
INR, median, median (s)	2.1	2.3	2.6	1.2	1.8	N/A
Platelet count, median (1000/μL)	194.9	205.0	187.3	261.6	175.7	N/A
Hemoglobin, median (g/dL)	11.2	10.2	9.2	13.4	13.2	N/A
Laboratory data after idarucizumab						
aPTT, median (s)	34.5	33.1	35	25.6	38.6	N/A
INR, median, median (s)	1.3	1.3	1.3	1.1	1.3	N/A

DOAC: direct oral anticoagulant; aPTT: activated partial thromboplastin time; INR: international normalized ratio. ^a^ Defined as normalization of the activated partial-thromboplastin time (aPTT) after idarucizumab infusion. ^b^ Defined as intra-hospital mortality, consisting of death by any cause, documented in the medical record, after idarucizumab infusion, during hospitalization. ^c^ Including all arterial and venous thromboembolic events. ^d^ Including adverse effects related to idarucizumab within 30 days.

**Table 2:** Real-world effectiveness of idarucizumab based on the major exclusion criteria of the REVERSE AD trial.

	Patients, *n* (%)	Hemostasis, *n* (%)	Post-Infusion Bleeding, *n* (%)	Thromboembolic or Adverse Events ^a^, *n* (%)	Mortality ^b^, *n* (%)
Total patients	32 (100%)	24/26 (92.3%)	1/26 (3.9%)	1 (3.1%)	5 (15.6%)
Ineligible patients	11 (34.3%)	4/5 (80%)	0/5 (0%)	0 (0%)	3/11 (27.3%)
Before TPA ^c^	5 (15.6%)	N/A	N/A	0/5 (0%)	0/21 (0%)
Did not take dabigatran	4 (12.5%)	3/4 (75%)	1/4 (25%)	0/4 (0%)	2/4 (50%)
Minor bleeding	1 (3.1%)	1/1 (100%)	0/1 (%)	0/1 (0%)	0/1 (0%)
Sepsis without active bleeding	1 (3.1%)	N/A	N/A	0/1 (0%)	1/1 (100%)
Eligible patients	21 (65.6%)	20/21 (95.2%)	1/21 (4.8%)	1/21 (4.8%)	2/21 (9.5%)

^a^ Included all arterial and venous thromboembolic events. ^b^ Defined as intra-hospital mortality, consisting of death by any cause, documented in the medical record, after idarucizumab infusion, during hospitalization. ^c^ TPA: tissue plasminogen activator.

**Table 3:** Baseline characteristics and clinical outcomes in study patients.

Patient Number	Gender (Age)	CCR, mL/min	DOAC Dosage	HASBLEED Score	CHA_2_DS_2_-VASc Score	Eligibility for REVERSE AD	Hemostasis	Thromboembolic or Adverse Effects	Mortality
Group A: uncontrollable or life-threatening bleeding									
1	Male (89)	21.2	Dabigatran, 150 mg, BID	4	4	Yes (GI bleeding)	Yes	No	Yes
2	Male (65)	40.1	Dabigatran, 150 mg, BID	5	4	Yes (ICH)	No	No	No
3	Female (92)	64.1	Dabigatran, 150 mg, BID	2	8	Yes (GIB)	Yes	No	No
4	Female (89)	31.3	Dabigatran, 150 mg, BID	3	5	Yes (GIB)	Yes	No	No
5	Male (68)	56.7	Dabigatran, 110 mg, BID	3	6	Yes (Compartment syndrome)	Yes	No	No
6	Female (84)	50.4	Dabigatran, 150 mg, BID	4	7	Yes (Compartment syndrome)	Yes	No	No
7	Female (85)	72.5	Dabigatran, 150 mg, BID	4	5	Yes (ICH)	Yes	No	No
8	Male, (87)	58.5	Dabigatran, 150 mg, BID	2	7	Yes (ICH)	Yes	No	No
9	Female (91)	34	Dabigatran, 150 mg, BID	4	6	Yes (Compartment syndrome)	Yes	No	No
10	Male (75)	82.4	Dabigatran, 150 mg, BID	2	3	Yes (ICH)	Yes	No	No
11	Male (76)	83.1	Dabigatran, 150 mg, BID	3	5	Yes (GIB)	Yes	No	No
12	Female (77)	14.25	Dabigatran, 110 mg, BID	6	6	Yes (Hemoptysis)	Yes	No	No
13	Female (88)	46.8	Dabigatran, 150 mg, BID	3	7	Yes (GIB)	Yes	No	No
14	Female (64)	6.2	Dabigatran, 110 mg, BID	2	2	Yes (GIB)	Yes	No	No
15	Male (82)	39.1	Dabigatran, 110 mg, BID	3	5	Yes (GIB)	N/A	No	Yes
16	Male (87)	91.4	Dabigatran, 150 mg, BID	1	5	Yes (GIB)	N/A	No	No
Group B: Emergency surgery or invasive procedures									
17	Female (92)	70.4	Dabigatran, 150 mg, BID	1	3	Yes (PPU)	Yes	Yes	No
18	Male, (70)	56.7	Dabigatran, 150 mg, BID	2	6	Yes (ICH)	Yes	No	No
19	Male (71)	56.9	Dabigatran, 150 mg, BID	3	4	Yes (ICH)	Yes	Yes	No
20	Female (71)	105.8	Dabigatran, 110 mg, BID	1	6	Yes (ICH)	Yes	No	No
21	Female (69)	79	Dabigatran, 150 mg, BID	2	6	Yes (ICH)	N/A	Yes	No
17	Female (92)	70.4	Dabigatran, 150 mg, BID	1	3	Yes (PPU)	Yes	Yes	No
Group C: Ineligible for clinical trials (RE-VERSE AD)									
22	Male (62)	51.5	Dabigatran, 150 mg, BID	2	6	NO (Pre-TPA/IA)	Yes	No	No
23	Female (63)	59.6	Dabigatran, 150 mg, BID	3	7	NO (Pre-TPA)	Yes	No	No
24	Female (70)	87.3	Dabigatran, 150 mg, BID	3	3	NO (Pre-TPA)	Yes	No	No
25	Male (53)	70	Dabigatran, 150 mg, BID	2	1	NO (Pre-TPA)	Yes	No	No
26	Male (91)	105.9	Dabigatran, 150 mg, BID	3	4	NO (Pre-TPA)	Yes	Yes	No
27	Male (79)	47.9	Rivaroxaban, 10 mg QD	2	2	NO (did not take dabigatran)	1	No	NO
28	Female (55)	89.1	Rivaroxaban, 1/4 10 mg BID	4	6	NO (did not take dabigatran)	1	No	Yes
29	Female (77)	90.22	Apixaban, 5 mg QD	2	5	NO (did not take dabigatran)	0	No	Yes
30	Male (64)	34.4	Rivaroxaban, 15 mg QD	0	1	NO (did not take dabigatran)	1	No	No
31	Female (87)	71.9	Dabigatran, 110 mg, BID	4	6	NO (Sepsis without active bleeding)	N/A	No	Yes
32	Female (64)	121	Dabigatran, 150 mg, BID	2	5	NO (minor bleeding)	1	No	No

## Data Availability

The data presented in this study are available on request from the corresponding author. The data are not publicly available due to the containing information that could compromise the privacy of research participants.

## References

[1] Connolly S.J., Ezekowitz M.D., Yusuf S., Eikelboom J., Oldgren J., Parekh A., Pogue J., Reilly P.A., Themeles E., Varrone J. (2009). Dabigatran versus Warfarin in Patients with Atrial Fibrillation. N. Engl. J. Med..

[2] Schulman S., Kakkar A.K., Goldhaber S.Z., Schellong S., Eriksson H., Mismetti P., Christiansen A.V., Friedman J., Le Maulf F., Peter N. (2014). Treatment of Acute Venous Thromboembolism with Dabigatran or Warfarin and Pooled Analysis. Circulation.

[3] Van der Wall S.J., Lopes R.D., Aisenberg J., Reilly P., van Ryn J., Glund S., Elsaesser A., Klok F.A., Pollack C.V., Huisman M.V. (2019). Idarucizumab for Dabigatran Reversal in the Management of Patients with Gastrointestinal Bleeding. Circulation.

[4] Levy J.H., van Ryn J., Sellke F.W., Reilly P.A., Elsaesser A., Glund S., Kreuzer J., Weitz J.I., Pollack C.V. (2019). Dabigatran Reversal with Idarucizumab in Patients Requiring Urgent Surgery: A Subanalysis of the RE-VERSE AD Study. Ann. Surg..

[5] Vene N., Mavri A., Božič-Mijovski M., Gregorič M., Uštar K.K., Žerjav U., Gradišek P., Stecher A., Frol S., Nedog V. (2020). Idarucizumab for dabigatran reversal in daily clinical practice: A case series. Eur. J. Anaesthesiol..

[6] Brennan Y., Favaloro E.J., Pasalic L., Keenan H., Curnow J. (2019). Lessons learnt from local real-life experience with idarucizumab for the reversal of dabigatran. Intern. Med. J..

[7] Majeed A., Hwang H.G., Connolly S.J., Eikelboom J.W., Ezekowitz M.D., Wallentin L., Brueckmann M., Fraessdorf M., Yusuf S., Schulman S. (2013). Management and Outcomes of Major Bleeding during Treatment with Dabigatran or Warfarin. Circulation.

[8] Schulman S., Kearon C., Kakkar A.K., Mismetti P., Schellong S., Eriksson H., Baanstra D., Schnee J., Goldhaber S.Z. (2009). Dabigatran versus Warfarin in the Treatment of Acute Venous Thromboembolism. N. Engl. J. Med..

[9] Reilly P.A., van Ryn J., Grottke O., Glund S., Stangier J. (2016). Idarucizumab, a Specific Reversal Agent for Dabigatran: Mode of Action, Pharmacokinetics and Pharmacodynamics, and Safety and Efficacy in Phase 1 Subjects. Am. J. Med..

[10] Yasaka M., Ikushima I., Harada A., Imazu S., Taniguchi A., Norris S., Gansser D., Stangier J., Schmohl M., Reilly P.A. (2017). Safety, pharmacokinetics and pharmacodynamics of idarucizumab, a specific dabigatran reversal agent in healthy Japanese volunteers: A randomized study. Res. Pr. Thromb. Haemost..

[11] Pollack C.V., Reilly P.A., van Ryn J., Eikelboom J.W., Glund S., Bernstein R.A., Dubiel R., Huisman M.V., Hylek E.M., Kam C.W. (2017). Idarucizumab for Dabigatran Reversal—Full Cohort Analysis. N. Engl. J. Med..

[12] Fanikos J., Murwin D., Gruenenfelder F., Tartakovsky I., França L.R., Reilly P.A., Kermer P., Wowern F.V., Lane D.A., Butcher K. (2020). Global Use of Idarucizumab in Clinical Practice: Outcomes of the RE-VECTO Surveillance Program. Thromb. Haemost..

[13] Thibault N., Morrill A.M., Willett K.C. (2018). Idarucizumab for Reversing Dabigatran-Induced Anticoagulation: A Systematic Review. Am. J. Ther..

[14] Syed Y.Y. (2016). Idarucizumab: A Review as a Reversal Agent for Dabigatran. Am. J. Cardiovasc. Drugs.

[15] Rodrigues A.O., David C., Ferreira J.J., Pinto F.J., Costa J., Caldeira D. (2020). The incidence of thrombotic events with idarucizumab and andexanet alfa: A systematic review and meta-analysis. Thromb. Res..

[16] Haastrup S.B., Hellfritzsch M., Nybo M., Hvas A.M., Grove E.L. (2020). Real-world experience with reversal of dabigatran by idarucizumab. Thromb. Res..

[17] Gómez-Outes A., Alcubilla P., Calvo-Rojas G., Terleira-Fernández A.I., Suárez-Gea M.L., Lecumberri R., Vargas-Castrillón E. (2021). Meta-Analysis of Reversal Agents for Severe Bleeding Associated with Direct Oral Anticoagulants. J. Am. Coll. Cardiol..

[18] Lu V.M., Phan K., Rao P.J., Sharma S.V., Kasper E.M. (2019). Dabigatran reversal by idarucizumab in the setting of intracranial hemorrhage: A systematic review of the literature. Clin. Neurol. Neurosurg..

[19] Cuker A., Burnett A., Triller D., Crowther M., Ansell J., Van Cott E.M., Wirth D., Kaatz S. (2019). Reversal of direct oral anticoagulants: Guidance from the Anticoagulation Forum. Am. J. Hematol..

[20] Barber P.A., Wu T.Y., Ranta A. (2020). Stroke reperfusion therapy following dabigatran reversal with idarucizumab in a national cohort. Neurology.

[21] Pikija S., Sztriha L.K., Sebastian Mutzenbach J., Golaszewski S.M., Sellner J. (2017). Idarucizumab in Dabigatran-Treated Patients with Acute Ischemic Stroke Receiving Alteplase: A Systematic Review of the Available Evidence. CNS Drugs.

[22] Kermer P., Eschenfelder C.C., Diener H.C., Grond M., Abdalla Y., Abraham A., Althaus K., Becks G., Berrouschot J., Berthel J. (2020). Antagonizing dabigatran by idarucizumab in cases of ischemic stroke or intracranial hemorrhage in Germany-Updated series of 120 cases. Int. J. Stroke.

[23] Giannandrea D., Caponi C., Mengoni A., Romoli M., Marando C., Gallina A., Marsili E., Sacchini E., Mastrocola S., Padiglioni C. (2019). Intravenous thrombolysis in stroke after dabigatran reversal with idarucizumab: Case series and systematic review. J. Neurol. Neurosurg. Psychiatry.

[24] Fang C.W., Tsai Y.T., Chou P.C., Chen H.M., Lu C.M., Tsao C.R., Chen C.L., Sun M.C., Shih Y.S., Hsieh C.Y. (2019). Intravenous Thrombolysis in Acute Ischemic Stroke after Idarucizumab Reversal of Dabigatran Effect: Analysis of the Cases from Taiwan. J. Stroke Cerebrovasc. Dis..

[25] Shahjouei S., Zand R. (2020). Response by Shahjouei and Zand to Letter Regarding Article, “Safety of Intravenous Thrombolysis among Patients Taking Direct Oral Anticoagulants: A Systematic Review and Meta-Analysis”. Stroke.

[26] Frol S., Sagris D., Pretnar Oblak J., Šabovič M., Ntaios G. (2021). Intravenous Thrombolysis after Dabigatran Reversal by Idarucizumab: A Systematic Review of the Literature. Front. Neurol..

[27] Shao S.C., Chan Y.Y., Kao Yang Y.H., Lin S.J., Hung M.J., Chien R.N., Lai C.C., Lai E.C. (2019). The Chang Gung Research Database—A multi-institutional electronic medical records database for real-world epidemiological studies in Taiwan. Pharmacoepidemiol. Drug Saf..

[28] Tsai M.S., Lin M.H., Lee C.P., Yang Y.H., Chen W.C., Chang G.H., Tsai Y.T., Chen P.C., Tsai Y.H. (2017). Chang Gung Research Database: A multi-institutional database consisting of original medical records. Biomed. J..

[29] Schulman S., Angerås U., Bergqvist D., Eriksson B., Lassen M.R., Fisher W. (2010). Definition of major bleeding in clinical investigations of antihemostatic medicinal products in surgical patients. J. Thromb. Haemost..

[30] Khorsand N., Majeed A., Sarode R., Beyer-Westendorf J., Schulman S., Meijer K. (2016). Assessment of effectiveness of major bleeding management: Proposed definitions for effective hemostasis: Communication from the SSC of the ISTH. J. Thromb. Haemost..

[31] Tomaselli G.F., Mahaffey K.W., Cuker A., Dobesh P.P., Doherty J.U., Eikelboom J.W., Florido R., Gluckman T.J., Hucker W.J., Mehran R. (2020). 2020 ACC Expert Consensus Decision Pathway on Management of Bleeding in Patients on Oral Anticoagulants: A Report of the American College of Cardiology Solution Set Oversight Committee. J. Am. Coll. Cardiol..

[32] Yasaka M., Yokota H., Suzuki M., Asakura H., Yamane T., Ogi Y., Ochiai K., Nakayama D. (2020). Idarucizumab for Emergency Reversal of Anticoagulant Effects of Dabigatran: Interim Results of a Japanese Post-Marketing Surveillance Study. Cardiol. Ther..

[33] Chaudhary R., Singh A., Chaudhary R., Bashline M., Houghton D.E., Rabinstein A., Adamski J., Arndt R., Ou N.N., Rudis M.I. (2022). Evaluation of Direct Oral Anticoagulant Reversal Agents in Intracranial Hemorrhage: A Systematic Review and Meta-analysis. JAMA Netw. Open.

[34] Jin C., Huang R.J., Peterson E.D., Laskowitz D.T., Hernandez A.F., Federspiel J.J., Schwamm L.H., Bhatt D.L., Smith E.E., Fonarow G.C. (2018). Intravenous tPA (Tissue-Type Plasminogen Activator) in Patients with Acute Ischemic Stroke Taking Non–Vitamin K Antagonist Oral Anticoagulants Preceding Stroke. Stroke.

[35] Calkins H., Hindricks G., Cappato R., Kim Y.H., Saad E.B., Aguinaga L., Akar J.G., Badhwar V., Brugada J., Camm J. (2018). 2017 HRS/EHRA/ECAS/APHRS/SOLAECE expert consensus statement on catheter and surgical ablation of atrial fibrillation: Executive summary. Europace.

[36] Calkins H., Willems S., Gerstenfeld E.P., Verma A., Schilling R., Hohnloser S.H., Okumura K., Serota H., Nordaby M., Guiver K. (2017). Uninterrupted Dabigatran versus Warfarin for Ablation in Atrial Fibrillation. N. Engl. J. Med..

[37] Di Biase L., Burkhardt J.D., Santangeli P., Mohanty P., Sanchez J.E., Horton R., Gallinghouse G.J., Themistoclakis S., Rossillo A., Lakkireddy D. (2014). Periprocedural stroke and bleeding complications in patients undergoing catheter ablation of atrial fibrillation with different anticoagulation management: Results from the Role of Coumadin in Preventing Thromboembolism in Atrial Fibrillation (AF) Patients Undergoing Catheter Ablation (COMPARE) randomized trial. Circulation.

[38] Zhao X., Chen L.Z., Su X., Long D.Y., Sang C.H., Yu R.H., Tang R.B., Bai R., Liu N., Jiang C.X. (2021). A strategy of idarucizumab for pericardial tamponade during perioperative period of atrial fibrillation ablation. Pacing Clin. Electrophysiol..

[39] Steffel J., Collins R., Antz M., Cornu P., Desteghe L., Haeusler K.G., Oldgren J., Reinecke H., Roldan-Schilling V., Rowell N. (2021). 2021 European Heart Rhythm Association Practical Guide on the Use of Non-Vitamin K Antagonist Oral Anticoagulants in Patients with Atrial Fibrillation. Europace.

[40] Okishige K., Yamauchi Y., Hanaki Y., Inoue K., Tanaka N., Yamaji H., Murakami T., Manita M., Tabata K., Ooie T. (2019). Clinical experience of idarucizumab use in cases of cardiac tamponade under uninterrupted anticoagulation of dabigatran during catheter ablation of atrial fibrillation. J. Thromb. Thrombolysis.

[41] Gragnano F., Calabrò P., Valgimigli M. (2019). Is triple antithrombotic therapy, or rather its duration and composition, the true culprit for the excess of bleeding events observed in patients with atrial fibrillation undergoing coronary intervention?. Eur. Heart J..

[42] Mourafetis J., Doctor N., Leung S. (2018). Treatment of gastrointestinal bleeding with idarucizumab in a patient receiving dabigatran. Am. J. Health Pharm..

[43] Kurdziel M., Hudzik B., Kazik A., Piegza J., Szkodziński J., Gąsior M. (2021). Idarucizumab for dabigatran reversal in cardiac tamponade complicating percutaneous intervention in ST elevation myocardial infarction. Adv. Interv. Cardiol..

